# Evidence‐Based Approaches to Quality Improvement: A Narrative Review of Integrating Bayesian Adaptive Trials Into Health Services

**DOI:** 10.1111/jep.70197

**Published:** 2025-07-06

**Authors:** Min Jung Kim, David Prieto‐Merino, Jennifer Nicholas, Luke Allen, Matthew J. Burton, Andrew Bastawrous, David Macleod

**Affiliations:** ^1^ Faculty of Epidemiology and Population Health London School of Hygiene & Tropical Medicine London UK; ^2^ Universidad de Alcalá Madrid Spain; ^3^ International Centre for Eye Health London School of Hygiene & Tropical Medicine (LSHTM) London UK

**Keywords:** adaptive clinical trial, bayesian analysis, health services research, implementation science, quality improvement

## Abstract

**Rationale:**

Quality improvement (QI) in health service programmes aims to make small, incremental changes to increase reach and efficiency. Simple, low‐risk programmatic changes can improve services, particularly when supported by robust evidence. However, in health service contexts, there is tension between the need for swift decision‐making and the high research standards for conducting methodologically rigorous trials. Randomized trials are rarely used to evaluate these changes due to high costs and long timelines, especially when the changes are expected to result in marginal improvements. Instead, health service programmes frequently introduce changes informed by anecdotal evidence or less robust evaluation methods such as before‐and‐after comparisons.

**Aims:**

In this paper, we present a narrative review of the concepts underlying Bayesian adaptive trial designs for conducting QI research, highlighting their use in the commercial sector and exploring opportunities for cross‐industry learning and future application in healthcare settings.

**Methods:**

Relevant studies were selected based on their contextual relevance to the topic, in keeping with the narrative review approach.

**Results:**

Given that programmatic changes typically yield modest improvements, we recommend that adaptive trial designs can strike a balance between obtaining reliable results and avoiding overly large sample sizes. We review how interim analysis and early stopping can be integrated into trials, allowing the level of rigour to be adjusted according to the proramme specifications.

**Conclusion:**

Adaptive trial designs hold significant promise for enhancing the QI efforts. To ensure that adaptive trial designs can be successfully integrated into health service contexts, tradeoffs should be made between methodological rigour and resource constraints.

## 1 Introduction

Quality improvement (QI) is a systematic approach aimed at enhancing the quality and delivery of care through incremental changes [1, 2], for example, by introducing simple, low‐risk initiatives such as incentives and reminders to improve adherence [3, 4]. For QI to be successful and sustainable, these programmatic changes must be carefully selected based on robust evidence of effectiveness [2, 5]. However, a gap often exists between evidence and practice. Many QI efforts rely on anecdotal evidence or before‐and‐after studies to assess the impact of programmatic changes [6, 7, 8]. Randomised controlled trials (RCTs) are rarely used due to their complexity and high costs, which presents a high barrier to entry for organisations that do not have sufficient statistical and research governance expertise in‐house. This is especially true when the expected impact of each programmatic change is modest [9], creating a tension between the need for reliable evidence and the practical constraints of conducting long, conventional RCTs within routine service delivery. Nonetheless, missed opportunities arise when there is insufficient evidence to distinguish between effective and ineffective changes. It has been asserted that ‘the absence of evidence is too costly, not the efforts to generate such evidence’ [2].

In commercial sectors, particularly in the online digital ecosystem, more flexible, efficient trial designs present opportunities for cross‐industry learning [10]. Leading companies perform online experiments, such as testing variations in webpage configurations, to optimize their services [11, 12, 13]. What facilitates these online experiments are their high throughput, low implementation costs, and the ability to observe outcomes almost instantaneously. These characteristics allow the incorporation of adaptive trial features, enabling continuous monitoring and adjustments that enhance the efficiency of testing. While adaptive trials remain relatively underutilized in health services [14], their increasing use across various areas of clinical research shows their potential as a viable testing method [15, 16, 17, 18, 19]. Given the success of these designs, there is a significant potential for extending their application into QI of health service programmes, fostering a learning health system that ‘captures data from practice, generates knowledge from the data and puts the knowledge back into practice to improve care’ [20].

This paper is a narrative review exploring the concepts of Bayesian adaptive trial designs and their potential application to drive QI in health service programmes. This review draws on a wide range of sources, including empirical studies, commentary, corporate blogs, and reports. By examining examples from both clinical and commercial sectors, this paper aims to inform the design of adaptive trials focusing on the following objectives: (a) evaluating programmatic changes that lead to even the smallest improvements in health service programmes; (b) obtaining meaningful, actionable evidence with relatively small sample sizes that are feasible in health service contexts; and (c) ensuring that the trials maintain sufficient reliability and accuracy to ensure patient safety.

## 2 Adaptive Trial Designs

### 2.1 What Are Adaptive Trials?

Adaptive trials are characterized by prespecified design features and decision criteria, allowing for flexibility to modify and tailor the trial during its course. Participants are initially randomised into different arms, with interim analyses of accumulating data guiding decisions on trial modifications. These modifications include early stopping, dropping or adding arms, adjusting the randomisation ratio, or changing the sample size, amongst other possibilities [21]. This flexibility gives researchers the opportunity to balance the trial's efficiency and reliability, depending on the research objectives.

**Figure 1:**
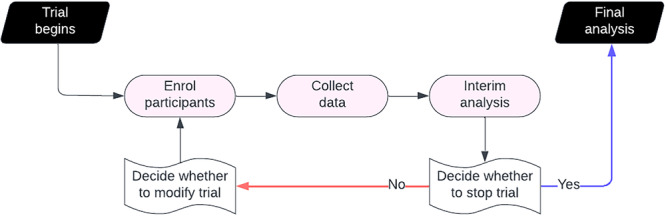
Adaptive trial processes. Adaptive trials begin by randomizing participants into trial arms and collecting data on how the programmatic change affects the outcome in the participants. At interim analysis, all data accumulated by this period is analysed to provide real‐time insights. Based on these results, decisions can be made to modify the trial. Interim analysis and modification repeat until a decision is made to stop the trial.

The decision to integrate adaptive design features into a trial is driven by the specific research goals it aims to achieve. For example, if the goal is to swiftly evaluate programmatic changes, stopping rules can be applied to terminate trials when early evidence demonstrates either efficacy or futility. Alternatively, the trial may continue to accrue additional data to strengthen the evidence. When the goal is to benefit trial participants through potentially promising programmatic changes, the trial can adjust allocation probabilities to favour changes that show the most potential or discontinue those that appear ineffective or harmful. These timely design adjustments can make trials more efficient and responsive, positioning adaptive designs as a promising approach for driving QI (Figure 1).

Researchers interested in adopting adaptive trials for QI research need to consider several factors. Commercial, online experiments are rapid because outcomes become available shortly after exposure to interventions, promptly guiding decision‐making [10]. For instance, in online marketing, the outcome of a visitor clicking on an advertisement can be observed almost instantaneously to provide real‐time insights into its effectiveness. Similarly, adaptive trials could be applied to QI in health services where the duration between intervention and outcome is relatively short, such as measuring attendance to referral appointments after receiving a reminder call. Having the capacity to analyze the data quickly as it becomes available is also essential for the efficient conduct of adaptive trials. Furthermore, the FDA emphasizes the importance of simulations in designing adaptive trials that warrant both feasible and acceptable parameters of sample sizes and error rates [17, 22]. However, since not all health services may have the resources or capacity to carry out simulations, there is a need for further research to establish comprehensive guidelines—outlining trial designs for testing various QI interventions—and provide a framework for broader adoption.

### 2.2 Bayesian Methods for Decision‐Making

Adaptive trial designs can utilize frequentist, Bayesian, or a hybrid of both approaches to estimate the impact of programmatic changes. Frequentist methods treat parameters as fixed and estimates the impact of changes based on the theory of repeated sampling. Bayesian methods, in contrast, view parameters as random quantities with distributions, which are continuously updated as new data accumulates. In this context, *belief* describes the degree of certainty about the parameter based on the distribution of posterior probability. The inherent ability to update belief complements the nature of continuous monitoring, making Bayesian methods well‐suited for adaptive trials [23].

Bayesian methods estimate the impact of programmatic changes using posterior probabilities, providing an intuitive interpretation that quantifies the credibility of the data and belief, along with their associated uncertainties [24, 25]. For example, conventional hypothesis testing using frequentist approach may state that there is an X% probability of observing a difference as extreme as the data between two variants of a programmatic change, assuming the null hypothesis of no difference is true. In contrast, a Bayesian method directly reports the probability, such as a Y% probability that one variant is better than the other, given the observed data. It generally offers a clearer and more straightforward interpretation, making Bayesian methods useful for informing health policy and clinical decision‐making [23, 26, 27, 28]. Similarly, in QI efforts, the use of Bayesian methods could support decision‐making by reporting the impact of programmatic changes in a more comprehensible manner.

Furthermore, Bayesian estimate provides an explicit representation of all available data up to the point of analysis as well as any prior beliefs. This is particularly beneficial when there are multiple perspectives and evidence available to inform decision‐making. Bayesian methods allow for diverse types and sources of information, such as expert knowledge and prior studies, to be integrated into a single estimate [29, 30]. This is different from frequentist framework, which typically relies on a pooled analysis of closely related studies.

Nonetheless, there are practical barriers associated with the use of Bayesian approaches. First, analysing data using Bayesian approaches often requires intensive computation and statistical support. For those who are more familiar with frequentist approaches, the inherent conceptual difference between the two methods adds a steep learning curve. Similarly, the elicitation of a prior distribution is often not a straightforward task. While existing information or expert opinion may inform the selection of a prior, translating various types of information into a single, coherent prior can be difficult. This becomes particularly difficult in Bayesian adaptive trials, where the prior significantly biases estimation and influences the decision‐making based on posterior distribution [31].

Several learning platforms and strategies have been made available to address these barriers and make Bayesian approaches more accessible to various audiences [24, 32, 33]. The following sections will provide discussions on leveraging adaptive designs for QI, focusing on Bayesian approaches.

### 2.3 Adaptive Design Features for Achieving Quality Improvement

#### 2.3.1 Selecting Prior Beliefs

In a Bayesian framework, posterior distributions represent the updated belief about a parameter, such as the impact of programmatic changes. Posterior distributions are created by combining prior distributions with observed data, representing the updated belief that accounts for both prior knowledge and new information. Therefore, an essential step towards planning a Bayesian adaptive trial is the pre‐specification of prior distributions, which can often have substantial impact on the resulting posterior distributions [30]. Ideally, priors should be carefully selected to align with both the objective of the trial and the intended use of its results [34].

Noninformative priors can be used when there is limited evidence or uncertainty about the impact of programmatic changes. These priors make minimal assumptions about the impact of the changes, allowing the posterior distribution to be solely shaped by the accumulating data [34]. This is advantageous when trial results are intended for consideration across various settings with different prior beliefs. Alternatively, a sceptical prior assumes that aa programmatic change has minimal or no benefit, and it requires the accumulating data to provide strong evidence of efficacy to overcome this scepticism and demonstrate its impact [23, 35]. For example, decisions on whether to introduce an expensive change should be based on robust evidence indicating its efficacy. Employing a sceptical prior sets a high bar justifying the cost implications and determining whether the evidence is reliable enough for the change to be integrated into the programme.

Another key advantage of using a Bayesian approach is its ability to incorporate informative priors. May QI efforts have utilized existing information—such as expert knowledge or evidence from similar settings—in less formal ways to decide whether it is worth adopting a change into a programme. Currently, there is no standardised method in QI research for incorporating such diverse types of evidence [36]. Bayesian methods provide a more robust statistical approach, with techniques like prior elicitation, to formally integrate expert knowledge and pre‐existing evidence into the analysis [29].

#### 2.3.2 Interim Analysis

Interim analysis is a crucial component in the design of adaptive trials, serving as designated checkpoints for assessing the accumulated data and making decisions. A well‐structured interim analysis plan specifies the timing or frequency of analyses [16]. In online, commercial examples, QI experiments are strategically designed to avoid premature analyses, preventing random fluctuations observed in the early data from influencing decision‐making. For example, Google Analytics had implemented a default minimum 2‐week run‐in period into their experimental framework before conducting the first interim analysis [37], and Etsy had a minimum 7‐day period to account for different trends in weekdays and weekends [38]. A similar example seen in clinical research is the STAMPEDE trial, a multi‐arm multi‐stage trial comparing therapy options for prostate cancer, which conducted interim analyses only after a fixed number of outcomes were observed in the control arm [39]. These approaches ensured that sufficient data were collected to make reliable decisions. Similarly, for QI efforts of health service programmes, it can be beneficial to include a run‐in period particularly when data collection is slow or intermittent. In cases when the speed of data accrual is uncertain, it would be preferable to specify the run‐in period in terms of sample size rather than a fixed period of time.

Furthermore, the timing and frequency of interim analyses should align with the goals of QI efforts. Frequent data analyses offer timely insights but at the cost of being operationally burdensome and increasing the risk of false positives results [40, 41]. For instance, Spotify conducts its experiments at prespecified intervals rather than continuously to reduce the risk of false positives [42]. Conversely, infrequent interim analysis can reduce the chance of false positives but lead to less timely decision‐making. In health service programmes, interim analysis should also be planned considering the balance between speed and accuracy, ensuring that it does not compromise the integrity nor feasibility of the trial.

#### 2.3.3 Decision Criteria

At interim analysis, decisions can be made to modify the trial design and declare the effects of programmatic changes. This process involves assessing all data collected until a specific interim analysis point against predefined decision criteria. For example, posterior distributions about the impact of programmatic changes can be compared to prespecified thresholds to determine whether sufficient evidence has been collected to make informed decisions about which change to adopt.

Amazon illustrates an example of a commercial QI effort where interim analysis results are assessed against decision criteria. They offer online experiment platforms for their sellers, enabling them to create and conduct experiments on various attributes—such as different variants of product images or descriptions—and evaluate the impact on sales or conversion rates. Sellers can evaluate their experiment results by monitoring the updated posterior probabilities each week and assess degree to which each variant is more effective than the other [13].

Another experimental platform, Google Analytics, also demonstrated the use of decision criteria to help online businesses optimise services. For example, experiments were conducted using Bayesian approaches to compare different variants of a webpage. At an interim analysis, a variant was identified as a winner if it had at least a 95% probability of being better. Conversely, if there was a 95% or higher probability that the top variants showed a negligible difference, the experiment terminated to conclude that these variants were virtually identical [37, 43].

**Figure 2:**
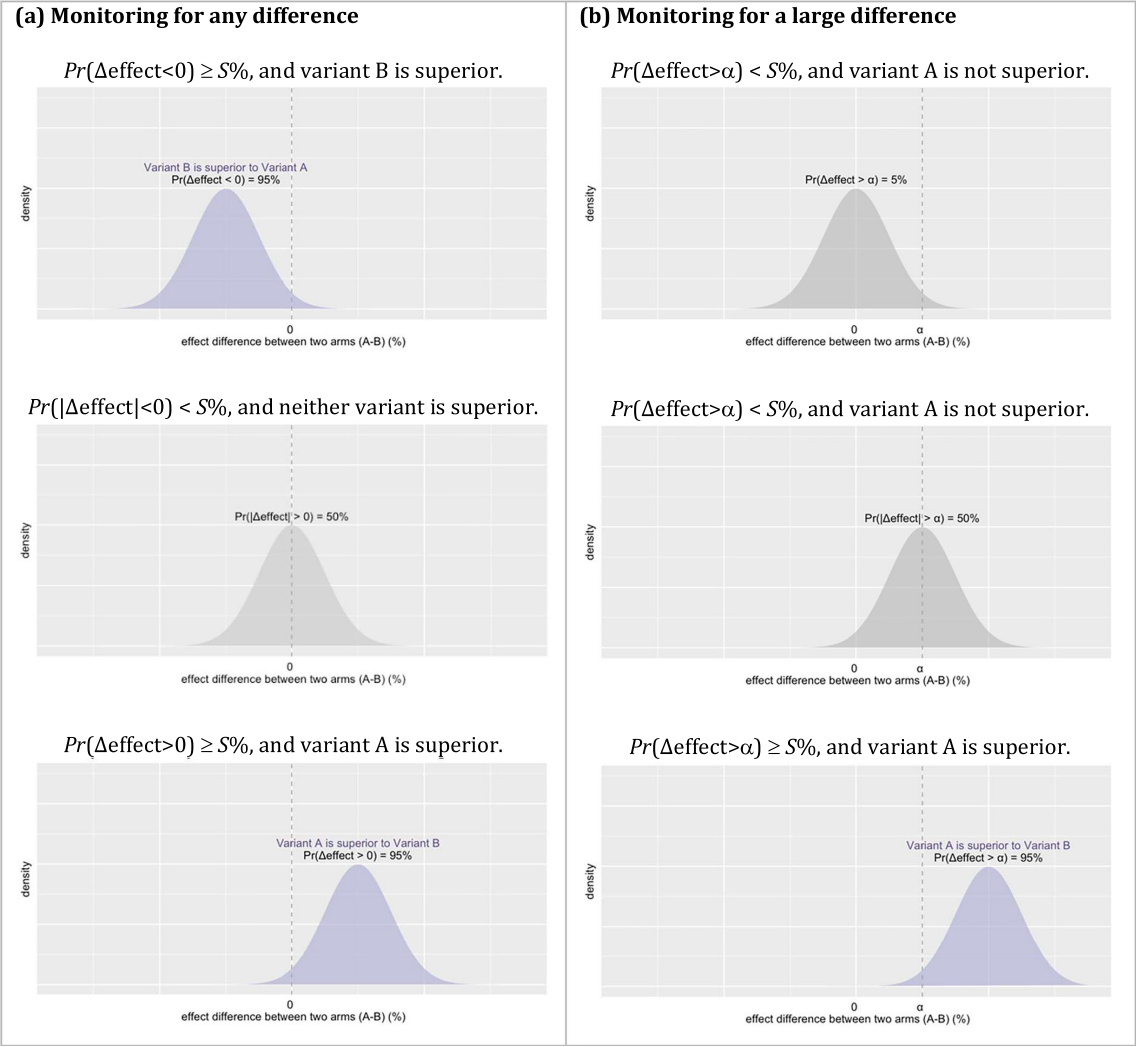
Constructing probability‐based decision criteria for identifying a superior variant. (a) An effect difference between two variants can be assessed by monitoring the posterior probability that the effect difference exceeds 0%. A variant can be identified as superior if this posterior probability is greater than a predefined threshold, such as *S* = 95%. (b) A larger effect difference can be identified by monitoring the posterior probability of the effect difference being greater than α. A variant can be identified as superior if this posterior probability exceeds a predefined threshold, such as *S* = 95%.

**Figure 3:**
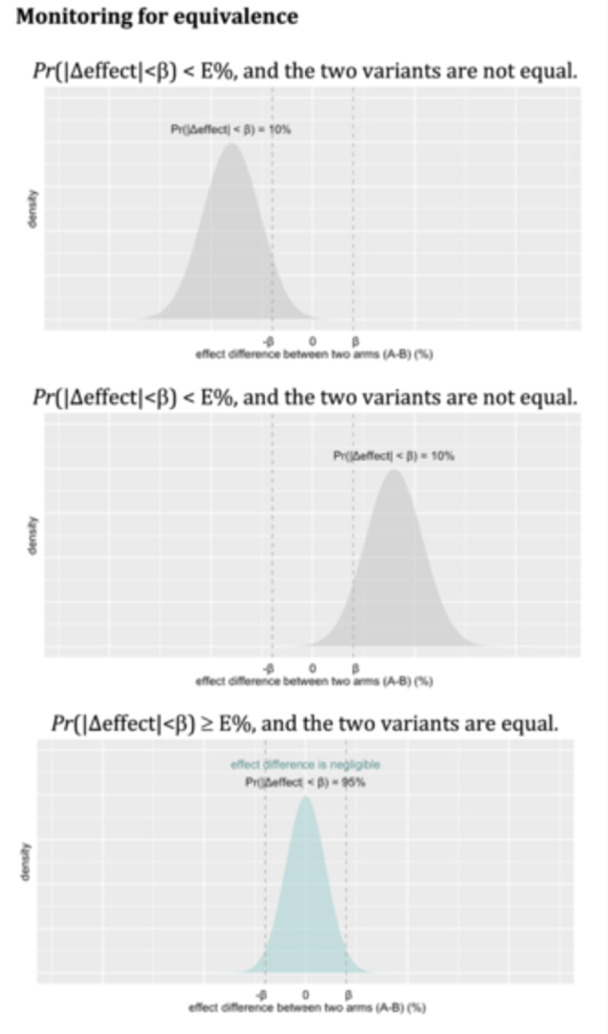
Constructing probability‐based decision criteria for identifying variants that perform equally. Equivalence between two variants can be assessed by monitoring the posterior probability that the effect difference between them is smaller than β. Equivalence can be declared if this posterior probability exceeds a predefined threshold, such as *E* = 95%.

Similarly, for QI efforts of health service programmes, decision criteria can be custom‐built to address specific needs, such as assessing whether a programmatic change results in improvement or harm, or determining when there is little value in continuing the trial to compare variants that perform similarly. In a Bayesian trial, the relative effect between two variants of a programmatic change can be represented as a posterior distribution. These distributions can then be used to calculate the posterior probability that the relative effect is greater than or smaller than a predefined threshold. Figures 2 and 3 illustrate examples of how such decision criteria can be established to assess the effect difference between two variants.

First, a superior variant can be identified by evaluating the posterior probability that it outperforms other variants. This involves assessing whether the probability is large enough to meet or exceed a predefined threshold *S* (*Pr*(|Δeffect| > α) ≥ *S*%). Here, Δeffect represents the effect difference between two variants (Δeffect = variant A–variant B), α is the minimum effect difference required for the two variants to be considered meaningfully different. *Pr* is the posterior probability that this effect difference is larger than α. If this *Pr* exceeds *S*, it indicates there is strong evidence to support one variant being superior to the other.

If α is set to 0, the decision criterion can be used to declare a variant as superior if there is a S % or higher posterior probability that the two variants are different by any amount. This is useful if the goal is to identify any variant that leads to a difference, even if it is marginal (Figure 2a). Alternatively, if α is set to a value greater than 0, a variant can be declared superior only if the effect difference between the superior variant and the inferior variant is larger than α. A large value of α would be useful in settings where one variant is more expensive than the other, and a large difference is needed to support the decision to select the more expensive, yet potentially more effective variant (Figure 2b).

Decisions can also be made based on the observation that the two variants of a programmatic change show similar effects. This can be assessed by evaluating the posterior probability that two variants have a negligible difference. If this probability exceeds a predefined threshold *E* (*Pr*(|Δeffect| < β) ≥ *E*%), it can be declared that the two variants perform very similarly. Here, β represents the maximum allowable difference in effect that is, deemed negligible, creating an ‘indifferent zone’ where the two variants are considered as being equal [35] (Figure 3). When equivalence is declared, decisions may be made to select a more appealing variant, taking into account factors like the cost and ease of integration.

To construct the decision criteria, it is important to select appropriate threshold values for defining efficacy or equivalence that are relevant to the goals and specifications of a programme. These decision thresholds would not only influence the final sample size but also the accuracy of the trial results. For such reasons, regulatory bodies such as the U.S. Food and Drug Administration (FDA) recommend that decision criteria be carefully established through simulations to ensure they align with the research objectives [17, 22]. In the context of QI efforts, decision criteria can be developed by considering both their feasibility within a given programme and the reliability of the trial results they generate.

#### 2.3.4 Optional Stopping

After assessing the accumulated data against the decision criteria, one possible decision to make is to stop the trial altogether. Implementing early stopping rules into a trial provides flexibility to stop when sufficient evidence has been collected to draw reliable conclusions or when continuing the trial beyond a certain point does not provide additional value. In many QI efforts where the anticipated benefit of the intervention is small or uncertain [2], the option to stop trial early can enhance efficiency by avoiding the need to commit to a fixed trial duration.

For QI efforts, early stopping can be advantageous for several reasons: when it becomes clear that continuing the trial is futile because there is no meaningful difference between variants; when a variant is clearly not effective enough to justify its cost; or if the primary goal is to quickly identify a promising variant, rather than precisely quantify the amount of the improvement. In adaptive trials, interim data can provide an early indication of an improvement, but it may not provide a precise estimate of the effect size because early stopping can lead to potential bias (this will be discussed further in Section 3.4) [44, 45, 46]. By incorporating stopping rules, researchers can choose to stop a trial early once a superior variant is identified, even if the exact magnitude of its impact is not certain.

The early stopping rules should be developed to strategically align with the trial's objectives. For example, therapeutic or drug trials commonly integrate stopping rules to prioritize patient safety. Pfizer's COVID‐19 vaccine efficacy trial included early stopping rules to immediately halt the trial if the vaccine showed potentially adverse effects, in accordance with the FDA authorization standards [47, 48]. Similarly, a remdesivir trial was designed to allow early stopping if interim analysis results indicated evidence of efficacy, futility, or safety concerns [49].

In contrast, QI efforts focused on low‐ or no‐risk programmatic changes can instead prioritize trial efficiency, ensuring that the trial can be successful integrated into existing programmes without overloading existing resources [2]. The strength of evidence to signal early stopping should be determined based on the programme needs and available resources. Researchers may choose to collect a moderate level of evidence if the goal is to quickly obtain results despite an increased risk of seeing errors or bias. Conversely, they may require stronger and more confident evidence for higher accuracy, especially when evaluating costly or high‐risk changes. Simulation studies can help guide the development of stopping rules that balance these trade‐offs between speed and accuracy [17, 22], ensuring that they align with the trial's objectives.

#### 2.3.5 Arm Dropping

When there are more than two variants to choose from, conducting multi‐arm trials can offer an efficient approach to simultaneously evaluate different variants. But assessing multiple variants through conventional, fixed‐size RCTs can be complex, especially when trying to maintain adequate statistical power with a limited amount of resources. Instead, incorporating adaptive design features, such as arm dropping, can be a responsive, efficient approach to reduce the overall sample sizes of trials. Many late‐phase trials introduced arm dropping features into their trial protocols to allow assessment of multiple treatments [50]. Based on predetermined decision rules, an arm can be discontinued when there is evidence of safety concerns, presence or lack of efficacy, or futility, thereby allowing the trial to continue with the remaining arms rather than stopping it completely.

Firstly, decisions can be made to drop unpromising variants from a trial. From a QI point of view, this approach can be beneficial as it redirects trial participants away from unpromising variants and towards more promising ones [51]. Alternatively, highly promising arms can graduate a trial early, as exemplified by the I‐SPY 2 trial for breast cancer drugs. In this example, enrolment to a treatment arm was stopped once sufficient evidence demonstrated the effectiveness of this treatment compared to the control and prompted this effective treatment arm to advance early to a phase III confirmatory trial. This decision rule was designed to accelerate the drug testing and approval processes [52]. In QI efforts, similar decisions can also be made to swiftly advance highly promising variants for broader adoption beyond trial participants.

Analysis methods must be planned carefully to accurately assess the effects of dropped arms relative the arms remaining in the trial. For instance, the STAMPEDE trial conducted a comparative analysis to assess every discontinued treatment arm against other arms that shared the same timeframes [53, 54]. The arm dropping strategy in the STAMPEDE trial is expected to evaluate eight treatment arms over 15 years, a process that would take about 40 years using conventional RCTs [55].

## 3 Evaluating the Impact of Adaptive Trial Features on Trial Integrity

While adaptive design features can make trials more flexible, they inevitably influence how trials perform under various conditions [16, 40, 56]. The tradeoff between sample size, accuracy, and bias should be managed carefully, ensuring that the benefits of adaptive design features outweigh their potential risks. For this reason, simulations are recommended as an ideal approach for evaluating the impact of these features [35, 40, 57, 58]. Evaluating adaptive design features and decision criteria across a range of plausible scenarios is the key to understand their true impact. The FDA emphasizes the use of simulations to estimate the expected error rates of a specific adaptive design before the trial begins [17, 22].

An optimal trial design should balance the tradeoff between accuracy and sample size to align with the trial's objectives. In pharmaceutical research, where patient safety is critical, the FDA advise early stopping only when there is high confidence in the findings or a compelling ethical rationale [17]. For QI of health service programmes, trial designs can be tailored to prioritize either higher accuracy or smaller sample sizes, depending on the context, such as resource availability and the nature of the programmatic changes. The balance between error rates and sample sizes should be adjusted according to which factor needs to be prioritised. Furthermore, trials should generate estimates that are accurate and reliable enough to inform the decisions about which programmatic changes to implement within a programme.

### 3.1 Sample Size

By incorporating early stopping rules in an adaptive trial design, it is possible to flexibly adjust the sample sizes, often requiring fewer participants than conventional RCTs [17, 18]. Evidence from the commercial sector illustrates that adaptive designs can be efficient for evaluating programmatic changes that have small impacts. However, these online experiments typically involve thousands of users as participants [59], allowing for error rates to be managed at desired levels. In contrast, not all health service programmes may have the advantage of such large sample sizes, making it essential to estimate the expected sample size and assess its feasibility within the specific programme context.

Simulations are a valuable tool for estimating the expected sample size of adaptive trial designs in a given context [17, 22, 35]. It helps estimate the average sample size needed to test programmatic changes with certain effect sizes. Robertson et al. showed that small‐sized trials are unlikely to stop early based on evidence of efficacy for a small intervention effect [46]. This makes it essential to evaluate whether an adaptive trial design of a certain sample size is sufficiently large enough to detect small effects. In cases where the effect sizes are uncertain, simulations can also replicate different scenarios with varying effect sizes to assess the average sample size across these different outcomes [60]. This involves analysing simulated datasets using the trial designs, evaluating whether the sample size predicted by these simulations is feasible within the available resources, and setting a maximum sample size to ensure the trial remains within practical limits.

### 3.2 Type I Error

In any research, it is crucial to establish target rates for both false positive (type I error) and false negative (type II error) rates. Using frequentist approaches, it is acknowledged that the type I error rate will increase with repeated testing of a single hypothesis or testing of multiple hypotheses. In Bayesian adaptive trials, however, the impact of interim analysis on the false positive rate remains a subject of debate. Some argue that Bayesian approaches are less susceptible to multiple testing problems due to their inherent nature of continuously updating the posterior distribution with accumulating data [35]. While some studies suggest that the false positive rates can be maintained within acceptable limits [61, 62, 63], others show that the false positive rates can be different depending on the type of priors used [40]. To better understand the error rate, it is recommended to leverage simulations to predict the risk of drawing false positive results using a given Bayesian adaptive design [40].

In clinical research, conventional RCTs are typically designed to maintain the type I error rate at or below 5%, either through a single final analysis or by adjusting the significance threshold at the final analysis to account for multiple analyses [17]. This is done to avoid mistakenly adopting ineffective or harmful treatments, which may result in adverse side effects or unnecessarily costs. Still, early phase clinical trials often allow a higher error rate to avoid prematurely discarding potentially effective treatments [64]. A more lenient error rate is also observed in oncology research studying rare cancers, where recruiting a large participant base is not possible. In this case, it has been demonstrated that conducting a series of shorter oncology trials with more relaxed error rates can be just as effective in improving survival rates as longer, more stringent trials [65, 66, 67].

In some QI efforts of health service programmes, false positives can have slightly different definitions compared to clinical research comparing an intervention against a control arm. First, a false positive may occur when a programmatic change that is not worse than others is mistakenly declared as most superior. Alternatively, one of several equally performing variants may be erroneously identified as more effective than the others. But in the latter case, particularly if all variants are low‐risk and similar in cost, the false positives of incorrectly declaring one variant as superior may not be considered harmful. However, when the programmatic changes involve additional costs or risks, it becomes increasingly important to focus on minimizing false positive rates.

### 3.3 Power

Well‐designed adaptive trials can achieve statistical power comparable to conventional fixed‐size RCTs [18]. In trials comparing two variants, power is defined as the likelihood of correctly identifying a superior variant when there is a true difference. Trials with low power carry the risk of failing to identify a superior variant. The potential harm of low power should be assessed for each programmatic change, considering its risk and cost.

When comparing more than two variants, multi‐arm adaptive trials can offer an efficient approach to streamline their evaluation [50]. In this context, power can take on different definitions depending on the trial's objectives. One such definition is conjunctive power, which refers to the likelihood of correctly assessing the performance of every variant in the trial [68]. It can be particularly relevant when the aim is to systematically compare the performance of multiple variants and identify the most promising one amongst all.

An alternative definition, marginal power, is the likelihood of correctly identifying a particular variant as superior amongst all other variants [68]. In QI efforts, assessing marginal power can be useful for comparing variants that have different costs. For example, marginal power can assess whether an expensive variant is worth its additional cost by demonstrating superiority over other cheaper alternatives.

Lastly, disjunctive power, is a more lenient definition focusing on identifying at least one superior variant, even if there are two or more equally superior arms [68]. It could be particularly relevant when the aim is to quickly identify any superior variant among all variants that have similar costs and safety concerns. In this case, researchers can prioritize identifying at least one superior variant as quickly as possible rather than investing resources to assess every single variant through pairwise comparisons.

### 3.4 Coverage and Bias

Well‐designed fixed‐size RCTs allow for unbiased estimation of effect sizes, and the analysis methods are usually relatively straightforward. In contrast, adaptive trials, which alters the design during their course, can introduce bias in effect size estimation. For example, early stopping may lead to an overestimation or overestimation of effect sizes [44, 45, 46]. When reporting the results of adaptive trials, it is essential to account for the adaptive design features used in the trial [17].

In the context of QI in health services, however, precise quantification of effect sizes may not be essential. Instead, QI efforts can focus on identifying the changes that lead to positive improvements, rather than evaluating the exact magnitude of those improvements. In the commercial sector, Etsy was an example of a company that acknowledged the risk of overestimation but opted to conduct adaptive trials to expedite the process of testing small‐impact changes [38]. In some cases, it may be sufficient to identify at least one promising variant or rank the variants based on their performance. Leveraging Bayesian approaches is particularly helpful in this context because they estimate the posterior probabilities that each variant outperforms the others, helping them assess the relative effects without the need to precisely quantify them.

## 4 Conclusion

QI is essential for optimizing health service programmes, enhancing both service delivery and patient outcomes. Many QI efforts have helped improve outcomes by introducing programmatic changes that are based on expert input or previous studies [36, 69]. To increase the odds of QI efforts achieving the intended goals, it is important to make evidence‐driven changes that are proven effective [2]. However, the use of randomised trials is often discouraged in QI of health service programmes, creating a disconnect between the need for robust evidence and the practical challenges of conducting conventional RCTs.

In this regard, the potential use of Bayesian adaptive trial designs is compelling for three key reasons. First, they offer a randomised trial approach for evaluating programmatic changes with modest effects, allowing flexible levels of methodological and statistical rigour that are appropriate for the context. Second, the trial design can be adapted to meet specific goals. For example, an adaptive trial design with early stopping rules can be useful when the aim is to quickly assess the relative effects of variants, without committing time and resources to measuring precise effect sizes. Lastly, Bayesian approaches could potentially enhance decision‐making by providing probability‐based outcome measures for intuitive interpretation of the trial results.

While Bayesian adaptive trials can offer a promising approach to drive QI of health service programmes, there are several practical challenges for implementing adaptive trials into real‐world health service settings. First, setting up adaptive trials can demand considerable effort and resources due to the need for interim analysis. The ability to conduct interim analysis is directly associated with the availability of accumulating data. It is therefore not suitable in settings where data is not readily available or of varying quality, which may limit the capacity to conduct interim analysis.

Frequent data analysis and adaptations can also demand considerable resources, both in terms of personnel and infrastructure. Trials should be integrated into the routine operations of a health service programme while minimizing disruption to ongoing services. Therefore, it is essential to ensure that health service programmes have adequate infrastructure and staffing to support the seamless integration of adaptive trials and maintain fidelity to the trial protocol.

Furthermore, simulation plays an important role in designing Bayesian adaptive trials that balance the efficiency and statistical rigour of the trial and tailored to achieve the specific research objectives [17, 22]. However, conducting simulations may be challenging or not feasible at all in some health service settings. Consideration must be given in the planning stage to determine whether the design and conduct of the Bayesian adaptive trials are viable.

Last but not least, requiring an ethics review for every programmatic change tested in an adaptive trial—specifically those that lead to marginal improvements, such as modifying the wording of reminder message—can introduce an additional bureaucratic layer. Nonetheless, when such changes pose minimal risk, the ethical concerns typically associated with clinical interventions are less applicable in this context, especially considering that many organizations are already making these types of changes without the formality of a research design. However, once these same changes are introduced through randomization, the line between routine QI efforts and scientific research begins to blur. Some studies have emphasized that QI research and traditional scientific research are worth distinguishing, particularly if the QI research is programmatic, involves minimal risk, and focuses on improving specific practices within a given context rather than generalizable knowledge [70, 71, 72].

While the findings presented in this review suggest that Bayesian adaptive trials hold potential to make QI efforts more efficient, it is important to acknowledge the methodological limitations of this narrative review. The inclusion of studies was not systematic, and potential selection and confirmation biases may have influenced the conclusions made. As such, the recommendations provided are exploratory in nature, and caution is warranted when making use of these discussions.

Further research, particularly with more rigorous methodologies, is needed to validate the insights presented in this review and confirm the potential strengths and limitations of using Bayesian adaptive trials to inform QI in health service settings.

## Conflicts of Interest

The authors declare no conflicts of interest.

## Data Availability

Data sharing not applicable to this article as no datasets were generated or analysed during the current study.
